# Physical activity and psychosocial profiles associated with mental health risk in community-dwelling older adults: a cross-sectional study

**DOI:** 10.3389/fspor.2026.1702851

**Published:** 2026-03-25

**Authors:** Cristina García, Lucrecia Moreno, Luis A. Martínez, Mónica Alacreu

**Affiliations:** 1 Cátedra DeCo MICOF-CEU UCH, Universidad Cardenal Herrera-CEU, Valencia, Spain; 2 Department of Pharmacy, Universidad Cardenal Herrera-CEU, CEU Universities, Valencia, Spain; 3 Department of Medical Sciences, School of Pharmacy, University of Castilla La Mancha (UCLM), Albacete, Spain; 4 Department of Mathematics, Physics and Technological Sciences, Universidad Cardenal Herrera-CEU, CEU Universities, Valencia, Spain

**Keywords:** mental health risk, older adults, physical activity, psychosocial factors, tailored exercise recommendations

## Abstract

**Introduction:**

Physical activity (PA) is widely studied in relation to mental health in older adults, yet the psychosocial factors that co-occur remain underexplored. This study explores associations between psychosocial profiles, PA levels, and screening-based mental health risk categories in community-dwelling older adults.

**Materials and methods:**

A cross-sectional study of 372 participants (mean age = 68.4 ± 7.2 years) assessed 34 variables across six domains to create comprehensive psychosocial profiles. Mental health risk was categorized into four levels based on depression and stress screening tools. Ordinal logistic regression examined associations between PA levels and mental health risk categories.

**Results:**

Mental health risk prevalence was 52% (95% CI: 46.4%–57.6%). Higher PA levels were associated with lower odds of higher mental health risk categories across age groups. Resilience and sense of coherence scores were higher among participants reporting moderate (vs. low) PA, whereas depressive symptom scores differed between low and high PA groups. Among the oldest-old individuals (≥85 years) the highest proportion of risk was observed in the low-PA group.

**Conclusion:**

The observed patterns suggest that PA, psychosocial factors, and mental health risk are interrelated and may vary by individual profile. These findings may inform hypothesis generation and the design of future longitudinal and intervention studies examining whether psychosocial profiling helps refine PA recommendations in routine care.

## 1 Introduction

Mental health conditions, particularly depression and anxiety disorders, represent a global epidemic among older adults, often characterized by symptoms that are challenging to manage and remain significantly undertreated (1). In Spain, the annual report of the National Health System indicates that 37% of the population experiences mental health conditions, with the prevalence increasing to as much as 50% in individuals over the age of 75 (2). In accordance with global trends, depression and psychological stress are the most diagnosed mood disorders. Depression has a significant impact on psychosocial functioning and on quality of life (3), while chronic psychological stress may contribute to mild cognitive decline and subsequent dementia (4).

Meta-analytic evidence supports the bidirectional association between stress and depression (5). This association is influenced by a set of biopsychosocial moderators and their interrelations that collectively determine an individual's capacity to cope with stressors throughout lifespan (6). In other words, the same stressors pose different risks depending on individual profiles. These determinants need to be addressed to raise coping thresholds. Among them, factors such as age, sex and genetics are not modifiable. Nevertheless, the modulation of psychosocial and life-style-related variables may play an important role in protecting individual well-being and has gained interest to the point of becoming a priority for tackling mental health improvement (7). Moreover, pharmacotherapy and psychotherapy remain underutilized and often yield modest effect sizes and high relapse rates (8, 9). Thus, implementing preventive non-pharmacological measures seems a key strategy to prevent the onset of stress or depression or once diagnosed, to complement pharmacological treatments outcomes and help control pathologies.

Data on psychosocial domain interventions for reducing symptoms and/or preventing depression and stress are available in the literature. Loneliness, a subjective emotional state resulting from a discrepancy between desired and actual social relationships (10), social isolation, an objective state characterized by a lack of social contacts and interactions with others (11), resilience, ability to adapt successfully in the face of adversity, trauma, or significant stress (12), and sense of coherence, reflecting perceptions of life as comprehensible, manageable, and meaningful (13), have been reviewed in this regard. Current evidence seems promising since some moderate effects have been reported; however, there remains considerable uncertainty regarding the implementation of any type of intervention in mental health services. In practice, it is difficult to design and develop direct interventions on psychosocial factors, and they can lead to issues related to participant privacy, autonomy, and respect (14).

Among the non-pharmacological lifestyle measures, regular physical activity (PA) is associated with the prevention and management of non-communicable chronic diseases. The 2020 World Health Organization guidelines recommend at least 150 min of moderate-intensity aerobic activity per week for older adults (15). There is evidence suggesting association between PA and mental health outcomes in depression, anxiety, and psychological stress-related disorders (16, 17) although the magnitude and directionality of this association remain to be determined (18). Individual PA level is associated with social interaction and improves self-rated well-being, which is linked to an increased likelihood of healthy aging (19, 20). The influence of psychosocial factors on the relationship between PA and mental health has recently been reviewed. Evidence supports key roles for self-esteem, affect, resilience, social support, and sense of control, highlighting the importance of psychological and social experiences as pathways through which PA benefits mental health (21).

The concept of ‘exercise as medicine’ is increasingly recognized worldwide, and precision health approaches emphasize tailoring lifestyle recommendations to individual characteristics. In this context, integrating psychosocial profiling with PA assessment may help describe which psychosocial profiles and mental health risk patterns are observed at different PA levels, providing a basis for future testing of tailored recommendations (22, 23).

We hypothesize that higher PA levels will be associated with lower mental health risk, and that these associations may differ across psychosocial profiles. The primary objective of this study is to characterize cross-sectional associations among PA, psychosocial factors, and mental health risk categories in community-dwelling older adults. Secondary objectives include exploring whether different PA levels are associated with different psychosocial factor distributions and describing a framework for prioritizing variables for future longitudinal or intervention work.

## 2 Materials and methods

### 2.1 Study and population

We conducted a cross-sectional study from January 2022 to September 2023. The inclusion criterion was non-institutionalized participants aged over 50. Exclusion criteria were participants diagnosed with Alzheimer's disease or dementia and participants with severe sensory, physical, or mental disability that would prevent their participation in an interview. The study's sample size (*n* = 372, 60% female; mean age = 68.4 ± 7.2 years) exceeds the minimum sample size (*n* = 318) required to estimate the percentage of participants at risk of stress and/or depression, considering a confidence level of 95% and an estimation error of 11%. The sample size meets commonly recommended requirements for logistic and ordinal regression models. Classic simulation studies indicate that at least 10 events per parameter are needed to obtain stable estimates and avoid bias in coefficients and confidence intervals (24, 25). These criteria are widely regarded as methodological standards for logistic models, including their ordinal extensions, with some authors advocating even more conservative thresholds depending on model complexity (26, 27). In our case, the ordinal logistic regressions use a four-category dependent variable and three coefficients for the proportional-odds predictors (age and two dummy variables for PA), meaning the model estimates approximately six parameters. Consequently, we have about 62 observations per parameter (372/6 ≈ 62) and at least 18 observations on the smaller side of the model's cumulative split at the highest threshold, thereby satisfying these conventional recommendations.

### 2.2 Data collection

Qualified pharmacists from our research group conducted face-to-face interviews in community pharmacies and health centers at appropriate facilities to ensure privacy. Interviewers were previously trained to acquire the necessary expertise and competencies to ensure the accurate and homogeneous administration of the assessment tool (28). The average duration of the interviews was 69 min, and they were structured into six sections: (a) sociodemographic factors, (b) cognitive impairment, (c) meaning in life, (d) psychosocial factors, including stress and depression, (e) health problems, and (f) lifestyle, including level of PA. The collected data comprised information concerning a total of 34 variables, which were subsequently entered into a spreadsheet for analysis. Further details on the measurements and assessment tools used are provided as Supplementary Table S1. The whole procedure has already been published (29).

#### 2.2.1 Mental health risk

The composite ordinal variable ‘mental health risk’ was constructed by assessing the presence or absence of risk of stress and depression using validated screening tools (30, 31). Consequently, participants were grouped into four categories: (i) no risk of stress nor depression, (ii) risk of stress only, (iii) risk of depression only, and (iv) risk of both stress and depression. This classification strategy was conceptualized based on the established evidence that these conditions represent a severity continuum with increasing functional impairment and psychosocial burden. The ordinal structure of the variable was modeled in the following way: absence of any condition represented optimal mental health, while the presence of a single condition indicated a significant deviation from baseline. Consequently, the comorbidity was designated as having the highest severity level. The rank ordering of the two single-condition categories (stress-only vs. depression-only) was determined based on the differential impact of each condition on global functioning. Depression is recognized as a leading cause of disability worldwide and produces more pervasive and persistent functional impairment than stress alone (32). Stress, in the absence of depression, may represent a subclinical or transitional state, often preceding the development of full-threshold mood disorders but not yet constituting the same severity of functional disruption (33). Accordingly, depression-only category was ranked above stress-only category in the ordinal scale. We acknowledge that this ordering constitutes a theoretical assumption that may not hold uniformly across all populations or measurement instruments.

Risk of stress was measured using the 4-item perceived stress scale (PSS-4) (34). The PSS-4 assesses the extent to which individuals have experienced situations as stressful during the preceding month. The participants responses were evaluated using a Likert scale (0 = ‘Never’; 4 = ‘Very often’) to measure their perceived stress levels. Higher scores indicated higher levels of perceived stress. Risk of depression and depressive symptoms were measured using the short 5-item version of the geriatric depression scale (GDS-5) (35). The GDS-5 is a suitable tool for assessing depression in older adults as it avoids questions related to physical symptoms and uses a straightforward ‘Yes’/’No’ format. A score greater than or equal to 2 indicates risk of depression.

#### 2.2.2 Physical activity

PA was measured using the short version of the international physical activity questionnaire validated for Spain (IPAQ-SF) (36). The IPAQ-SF consists of a brief, 7-item instrument designed to estimate an individual's level of PA over the past seven days. It collects information on the frequency and duration of walking, moderate, and vigorous physical activities, as well as time spent sitting. These data are used to calculate an estimate of PA expressed in metabolic equivalents (METs), which quantify the energy expenditure associated with different activity intensities. By converting reported activity into MET-minutes per day, the IPAQ-SF offers a standardized measure of PA that supports comparison across individuals and groups.

### 2.3 Transformation into zero-to-ten scale and data processing

To facilitate comparisons across variables assessed with different validated instruments, we first identified each instrument's minimum and maximum scores, and the validated cut-off points reported in the original validation studies (references provided in Supplementary Table S1). Original scales were then transformed to a common 0–10 metric using a linear min–max rescaling. This procedure preserves the ordering of observations and the proportional distances between scores and therefore does not alter the underlying psychometric properties of the original scales. Cut-offs defining the risk (red), intermediate (yellow), and protective (green) segments were determined by applying the same transformation to each instrument's validated thresholds, ensuring that the categorical bands on the 0–10 scale correspond exactly to established categories on the original scales rather than to sample-derived criteria. Color coding was used solely for descriptive visualization (see Table 1). Primary analyses were conducted on the continuous transformed scores to minimize information loss associated with categorization.

**Table 1:** Transformation of the original scales into zero-to-ten scales.

Variable blocks	Quantitative variable [original scale]	Group [original scale]	Formula	Group [10 scale]^a^
Meaning in life	Sense of coherence [13, 91]	Low [13, 64)	10Scale = (OriginalScale-13)/7.8	Low [0, 6.5)
		Intermediate [64, 80)		Intermediate [6.5, 8.6)
		High [80, 91]		High [8.6, 10]
	Purpose in life [6, 36]	No groups	10Scale = (OriginalScale-6)/3	No groups
Psychosocial factors	Resilience [4, 20]	Low [4, 13)	10Scale = (OriginalScale-4)/1.6	Low [0, 5.6)
		Intermediate [13, 17]		Intermediate [5.6, 8.1]
		High (17, 20]		High (8.1, 10]
	Stress [0, 16]	No risk [0, 5.4]	10Scale = OriginalScale/1.6	No risk [0, 3.4]
		Risk (5.4, 16]		Risk (3.4, 10]
	Depression [0, 5]	No risk [0, 2)	10Scale = 2*OriginalScale	No risk [0, 4)
		Risk [2, 5]		Risk [4, 10]
	Psychological distress [0, 12]	None [0, 2]	10Scale = OriginalScale/1.2	None [0, 1.7]
		Mild [3, 5]		Mild [2.5, 4.2]
		Moderate [6, 8]		Moderate [5, 6.7]
		Severe [9, 12]		Severe [7.5, 10]
	Social isolation [0, 30]	Social isolation [0, 12]	10Scale = OriginalScale/3	Social isolation [0, 4]
		No social isolation (12, 30]		No social isolation (4, 10]
Lifestyle	Body mass index (kg/m^2^)	Normal weight [18.5, 25)	10Scale = (OriginalScale-18.5)/2.15	Normal weight [0, 3)
		Overweight [25, 30)		Overweight [3, 5.3)
		Obese [30, +∞)		Obese [5.3, 10)
	Cognitive reserve [0, 25]	Low [0, 6]	10Scale = OriginalScale/2.5	Low [0, 2.4]
		Intermediate–low [7, 9]		Intermediate–low [2.8, 3.6]
		Intermediate–high [10, 14]		Intermediate–high [4, 5.6]
		High [15, 25]		High [6, 10]
	MeDAS [0, 14]	Low [0, 6]	10Scale = OriginalScale/1.4	Low [0, 4.3]
		Intermediate [7, 9]		Intermediate [5, 6.4]
		High [10, 14]		High [7.1, 10]

MeDAS, Mediterranean diet adherence screener.

^a^ Color coding of the transformed scales reflects the protective or risky nature of the scores.

Several of the study's variables can be characterized numerically and categorically. To assess the statistical associations with mental health risk, age, sense of coherence, resilience, psychological distress, and loneliness, among others, were treated qualitatively. In this case, the numerical intervals corresponding to each category are presented (Table 2, see Section 3 Results). When comparing the means as a function of the level of PA, these variables were treated as quantitative and the previously described color coding was used.

**Table 2:** Distribution of participants according to the mental health risk categories and the studied variables.

Group		Totals	i. Lower mental health risk	ii. Stress risk	iii. Depression risk	iv. Stress & Depression risk	*p*-value
		*n* (%)	*n* = 178 *n* (%)	*n* = 97 *n* (%)	*n* = 43 *n* (%)	*n* = 54 *n* (%)	
Sociodemographic factors							
Age	[50, 59]	45 (12.2)	25 (14.2)	13 (13.4)	1 (2.3)	6 (11.1)	0.030^a^^,^*
	[60, 69]	89 (24.1)	41 (23.3)	31 (32.0)	6 (14.0)	11 (20.4)	
	[70, 79]	122 (33.0)	63 (35.8)	29 (29.9)	15 (34.9)	15 (27.8)	
	[80, 97]	114 (30.8)	47 (26.7)	24 (24.7)	21 (48.8)	22 (40.7)	
Sex	Women	239 (64.4)	110 (62.1)	58 (59.8)	32 (74.4)	39 (72.2)	0.202^b^
	Men	132 (35.6)	67 (37.9)	39 (40.2)	11 (25.6)	15 (27.8)	
Marital status	Married	223 (60.3)	113 (63.8)	69 (71.9)	14 (32.6)	27 (50.0)	<0.001^a^^,^***
	Widow	110 (29.7)	46 (26.0)	20 (20.8)	22 (51.2)	22 (40.7)	
	Separated/divorced	20 (5.4)	9 (5.1)	4 (4.2)	2 (4.7)	5 (9.3)	
	Single	17 (4.6)	9 (5.1)	3 (3.1)	5 (11.6)	0	
Cognitive impairment							
Cognitive Impairment risk	Yes	71 (19.1)	32 (18.0)	15 (15.5)	12 (27.9)	12 (22.2)	0.324^b^
	No	301 (80.9)	146 (82.0)	82 (84.5)	31 (72.1)	42 (77.8)	
Meaning in Life							
Sense of coherence [13, 91]	Low [13, 64)	113 (30.5)	22 (12.4)	31 (32.3)	20 (46.5)	40 (75.5)	<0.001^a^^,^***
	Intermediate [64, 80)	189 (51.1)	99 (55.6)	58 (60.4)	22 (51.2)	10 (18.9)	
	High [80, 91]	68 (18.4)	57 (32.0)	7 (7.3)	1 (2.3)	3 (5.7)	
Psychosocial factors							
Resilience [4, 20]	Low [4, 13)	50 (13.4)	13 (7.3)	12 (12.4)	8 (18.6)	17 (31.5)	<0.001^b^^,^***
	Intermediate [13, 17]	208 (55.9)	89 (50.0)	64 (66.0)	25 (58.1)	30 (55.6)	
	High (17, 20]	114 (30.6)	76 (42.7)	21 (21.6)	10 (23.3)	7 (13.0)	
Psychological distress [0, 12]	None [0, 2]	198 (53.4)	132 (74.2)	41 (42.7)	17 (39.5)	8 (14.8)	<0.001^a^^,^***
	Mild [3, 5]	111 (29.9)	38 (21.3)	41 (42.7)	13 (30.2)	19 (35.2)	
	Moderate [6, 8]	45 (12.1)	7 (3.9)	14 (14.6)	10 (23.3)	14 (25.9)	
	Severe [9, 12]	17 (4.6)	1 (0.6)	0	3 (7.0)	13 (24.1)	
Loneliness [3, 9]	No loneliness [3, 6)	304 (88.1)	151 (93.8)	85 (95.5)	31 (73.8)	37 (69.8)	<0.001^b^^,^***
	Loneliness [6, 9]	41 (11.9)	10 (6.2)	4 (4.5)	11 (26.2)	16 (30.2)	
Social isolation [0, 30]	Social isolation [0, 12]	41 (11.0)	19 (10.7)	5 (5.2)	6 (14.0)	11 (20.4)	0.035^b^^,^*
	No social isolation (12, 30]	331 (89.0)	159 (89.3)	92 (94.8)	37 (86.0)	43 (79.6)	
Health problems							
Hypertension	Yes [systolic BP > 140 mmHg–diastolic BP > 90 mmHg]	219 (59.2)	103 (57.9)	54 (56.8)	27 (62.8)	35 (64.8)	0.733^b^
	No [systolic BP 140 mmHg–diastolic BP 90 mmHg]	151 (40.8)	75 (42.1)	41 (43.2)	16 (37.2)	19 (35.2)	
Hypercholesterolemia	Yes [CT > 200 mg/dL–CLDL > 100 mg/dL–CHDL 35–40 mg/dL]	170 (46.1)	74 (41.8)	44 (45.8)	28 (65.1)	24 (45.3)	0.055^b^
	No [CT 200 mg/dL–CLDL 100 mg/dL–CHDL > 35–40 mg/dL]	199 (53.9)	103 (58.2)	52 (54.2)	15 (34.9)	29 (54.7)	
Diabetes	Yes [blood glucose >126 mg/dL–HbA1c > 6.5%]	87 (23.5)	42 (23.6)	20 (20.6)	11 (25.6)	14 (26.4)	0.849^b^
	No [blood glucose 126 mg/dL–HbA1c < 6.5%]	284 (76.5)	136 (76.4)	77 (79.4)	32 (74.4)	39 (73.6)	
Smoking habit	Non-smoker	210 (56.6)	98 (55.1)	54 (56.2)	27 (62.8)	31 (57.4)	0.932^a^
	Former smoker	100 (27.0)	46 (25.8)	28 (29.2)	12 (27.9)	14 (25.9)	
	Smoker	40 (10.8)	22 (12.4)	10 (10.4)	3 (7.0)	5 (9.3)	
	Passive smoker	21 (5.7)	12 (6.7)	4 (4.2)	1 (2.3)	4 (7.4)	
Risk cardiovascular disease [1, 84]	Low (1, 5)	1 (0.3)	1 (0.6)	0	0	0	0.142^a^
	Mild [5, 9]	56 (15.1)	30 (17.0)	20 (20.6)	1 (2.3)	5 (9.3)	
	Moderate [10, 14]	32 (8.6)	12 (6.8)	8 (8.2)	4 (9.3)	8 (14.8)	
	Moderate–high [15, 19]	46 (12.4)	19 (10.8)	15 (15.5)	6 (14.0)	6 (11.1)	
	High [20, 29]	73 (19.7)	38 (21.6)	20 (20.6)	5 (11.6)	10 (18.5)	
	Very high [30, 84]	162 (43.8)	76 (43.2)	34 (35.1)	27 (62.8)	25 (46.3)	
Dependency	Yes	85 (22.8)	26 (14.6)	22 (22.7)	11 (25.6)	26 (48.1)	<0.001^b^^,^***
	No	287 (77.2)	152 (85.4)	75 (77.3)	32 (74.4)	28 (51.9)	
Chronic pain [0, 10]	No pain [0]	66 (23.1)	44 (30.8)	13 (18.8)	6 (16.7)	3 (7.9)	0.005^a^^,^**
	Mild [1, 3]	66 (23.1)	39 (27.3)	15 (21.7)	7 (19.4)	5 (13.2)	
	Moderate [4, 6]	102 (35.7)	45 (31.5)	28 (40.6)	11 (30.6)	18 (47.4)	
	Severe [7, 8]	41 (14.3)	11 (7.7)	10 (14.5)	10 (27.8)	10 (26.3)	
	Excruciating [9, 10]	11 (3.8)	4 (2.8)	3 (4.3)	2 (5.6)	2 (5.3)	
Lifestyle							
Body mass index (kg/m^2^)	Normal weight [18.5, 25)	108 (29.9)	53 (30.6)	37 (38.9)	5 (11.9)	13 (25.5)	0.012^a^^,^*
	Overweight [25, 30)	164 (45.4)	78 (45.1)	43 (45.3)	19 (45.2)	24 (47.1)	
	Obese [30, +∞]	89 (24.7)	42 (24.3)	15 (15.8)	18 (42.9)	14 (27.5)	
Retired	Yes	262 (70.8)	125 (70.2)	62 (63.9)	38 (88.4)	37 (71.2)	0.034^a^^,^*
	No	108 (29.2)	53 (29.8)	35 (36.1)	5 (11.6)	15 (28.8)	
Cognitive reserve [0, 25]	Low [0, 6]	76 (20.5)	32 (18.0)	17 (17.5)	12 (27.9)	15 (28.3)	0.031^a^^,^*
	Intermediate–low [7, 9]	75 (20.2)	38 (21.3)	14 (14.4)	15 (34.9)	8 (15.1)	
	Intermediate–high [10, 14]	104 (28.0)	52 (29.2)	28 (28.9)	11 (25.6)	13 (24.5)	
	High [15, 25]	116 (31.3)	56 (31.5)	38 (39.2)	5 (11.6)	17 (32.1)	
MeDAS [0, 14]	Low [0, 6]	44 (12.0)	17 (9.6)	9 (9.6)	9 (22.0)	9 (16.7)	0.220^b^
	Intermediate [7, 9]	159 (43.4)	76 (42.9)	40 (42.6)	19 (46.3)	24 (44.4)	
	High [10, 14]	163 (44.5)	84 (47.5)	45 (47.9)	13 (31.7)	21 (38.9)	
Physical activity	Low	96 (25.8)	38 (21.3)	22 (22.7)	16 (37.2)	20 (37.0)	0.030^a^^,^*
	Moderate	176 (47.3)	86 (48.3)	46 (47.4)	23 (53.5)	21 (38.9)	
	High	100 (26.9)	54 (30.3)	29 (29.9)	4 (9.3)	13 (24.1)	
MNA [0, 14]	Risk of malnutrition [0, 12)	128 (34.6)	53 (29.8)	36 (37.5)	7 (16.7)	32 (59.3)	<0.001^b^^,^***
	Normal nutrition [12, 14]	242 (65.4)	125 (70.2)	60 (62.5)	35 (83.3)	22 (40.7)	
Sleep [0, 20]	Sleep disorder [12, 20]	80 (21.5)	29 (16.3)	16 (16.5)	13 (30.2)	22 (40.7)	<0.001^b^^,^***
	No sleep disorder [0, 12)	292 (78.5)	149 (83.7)	81 (83.5)	30 (69.8)	32 (59.3)	
STOP-Bang [0, 8]	Low [0, 2]	147 (39.6)	75 (42.1)	43 (44.8)	14 (32.6)	15 (27.8)	0.107^b^
	Intermediate [3, 4]	160 (43.1)	77 (43.3)	39 (40.6)	16 (37.2)	28 (51.9)	
	High [5, 8]	64 (17.3)	26 (14.6)	14 (14.6)	13 (30.2)	11 (20.4)	
Group activity	Yes	133 (35.8)	75 (42.1)	34 (35.1)	12 (27.9)	12 (22.2)	0.035^b^^,^*
	No	239 (64.2)	103 (57.9)	63 (64.9)	31 (72.1)	42 (77.8)	

^a^ Fisher exact test.

^b^ Chi square test.

* *p*-value < 0.05.

** *p*-value < 0.01.

*** *p*-value < 0.001.

### 2.4 Statistical analysis

Statistical inference was carried out using R software (R-4.4.1, R Foundation for Statistical Computing, Vienna, Austria). The percentage of participants at risk of stress and/or depression was estimated (95% confidence interval). The association between participants’ mental health risk and the qualitative variables defining their profile was analyzed (Fisher's exact test and chi-squared test). An ordinal logistic regression model was fitted to estimate whether psychosocial variables vary systematically across PA levels and mental health categories. The Brant test indicated a partial violation of the proportional-odds assumption for age. We therefore conducted sensitivity analyses using a partially proportional odds specification. The ordinal (proportional-odds) model was retained for parsimony, as conclusions were qualitatively robust and model fit did not materially improve under more complex specifications. Statistically significant differences between the mean values of the quantitative variables as a function of the level of PA were analyzed (ANOVA and Tukey's multiple comparisons).

### 2.5 Ethical approval

This study was approved by the Biomedical Research Ethics Committee of the Universidad CEU Cardenal Herrera (approval no. CEI22/249). The data were treated confidentially and lawfully and used only for the purposes previously communicated to the participants. All the participants provided their written informed consent in accordance with the Declaration of Helsinki. Thus, this study complies with the European General Data Protection Regulation (GDPR) and Organic Law 3/2018 on the Protection of Personal Data and Guarantee of Digital Rights.

## 3 Results

The sample analyzed consisted of 372 individuals. The estimated percentage of participants at risk for stress and/or depression was 52% (95% confidence interval: [46.4, 57.6]). Table 2 summarizes the distribution of participants across the four mental health risk categories, along with a descriptive analysis of the variables that define participants’ profiles. Statistically significant associations with participants’ mental health risk were found for 16 of the 25 variables, suggesting that some profiles may be associated with certain mental health risk categories. On the one hand, 83.8% of the participants with a high sense of coherence, 66.7% of the participants with no pain, high resilience or no psychological distress exhibited a lower mental health risk (category 1). On the other hand, 76% of the participants with severe psychological distress, 39% of the participants who felt lonely, 35.4% of the participants with a low sense of coherence, 34% of the participants with low resilience, and 30.6% of the dependent participants were simultaneously at risk of stress and depression (category 4). Similarly, the percentage of the participants with a concurrent risk of stress and depression was 3.2-fold higher among those feeling lonely (39% vs. 12.2%), 2.7-fold higher for those who were malnourished (25% vs. 9.1%), 2.5-fold higher among those with sleep problems (27.5% vs. 11%), 2.1-fold higher among those being isolated (26.8% vs. 13%), 1.6-fold higher among those with low PA (20.8% vs. 13%), and 1.3-fold higher for those who were obese (15.7% vs. 12%).

**Figure 1:**
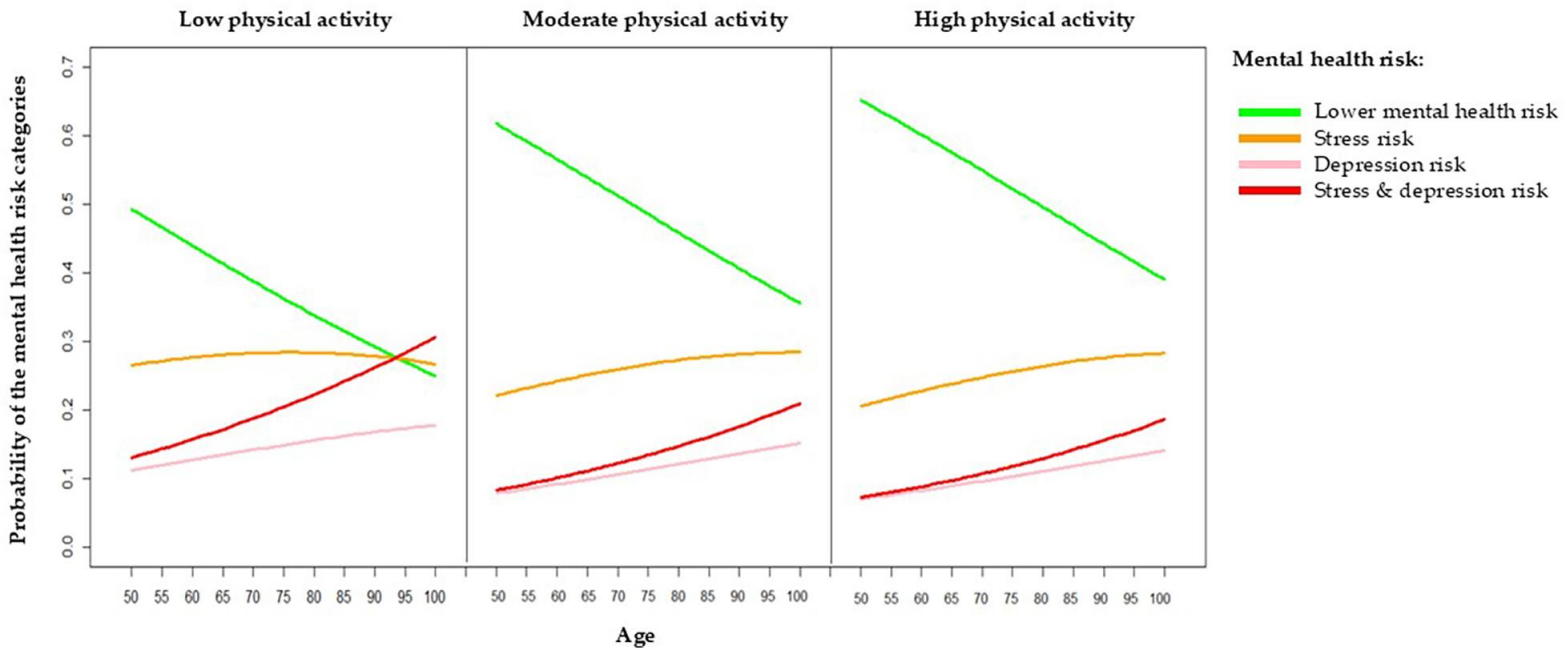
Distribution of mental health risk categories by age group and physical activity level. Colors indicate mental health risk: green = no risk, orange = stress only, pink = depression only, red = combined stress and depression.

Table 3; Figure 1 present the ordinal logistic regression model used to estimate the probability of each mental health risk category as a function of participant age and PA. Figure 1 presents the distribution of mental health risk categories across age groups and PA levels, providing a descriptive overview of the observed patterns. Higher PA levels are consistently associated with lower mental health risk across all age groups, with the strongest association observed among oldest-old participants, aged ≥85 years: only individuals with low PA are more likely to be classified as stressed or depressed, rather than having a low mental health risk.

**Table 3:** Ordinal logistic regression model.

	*β*_i_	SE	t value	*p*-value	Exp(β_i_)	95% CI	
						Lower limit	Upper limit
Age	0.021	0.009	2.286	0.022*	1.020	1.000	1.040
Physical activity [Moderate]	−0.507	0.236	−2.145	0.032*	0.603	0.379	0.957
Physical activity [High]	−0.654	0.271	−2.417	0.016*	0.520	0.305	0.882

βi, model coefficients; SD, standard error of the coefficients; Exp(βi), odds ratio; IC, confidence interval for the expected odds ratio.

* *p*-value < 0.05.

**Figure 2:**
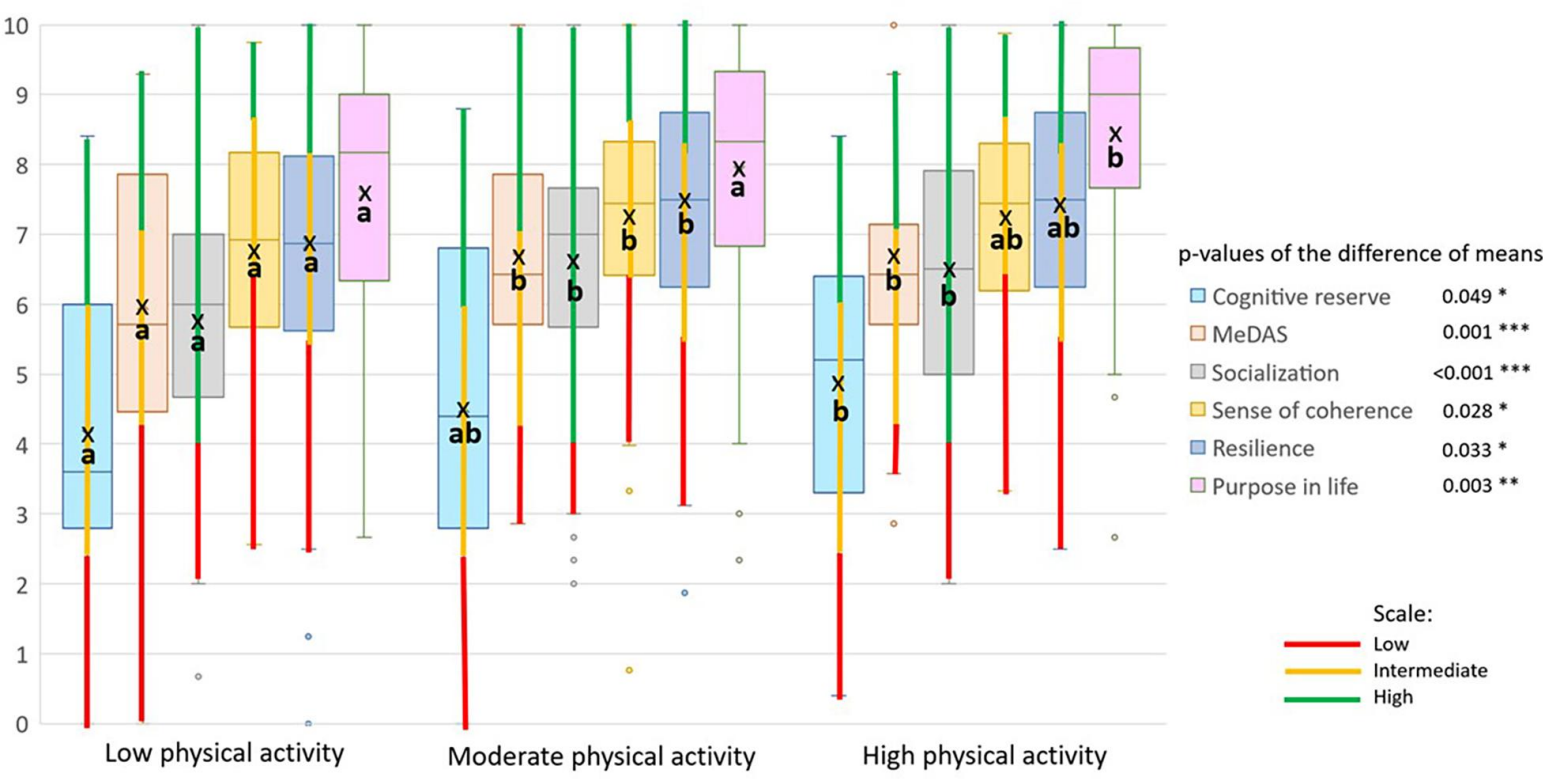
Box plots of the variables with direct association with physical activity (low, moderate, or high), represented in a zero-to-ten scale. The categories of each scale are shown along the whisker, using the same color coding presented in Table 1. Means are represented by ‘x’ inside the box (ANOVA *p*-values: *: *p*-value < 0.05; **: *p*-value < 0.01; ***: *p*-value < 0.001). In Tukey's multiple comparisons, ‘a' is significantly less than ‘b,' and ‘ab' is not significantly different to either ‘a' or ‘b’.

**Figure 3:**
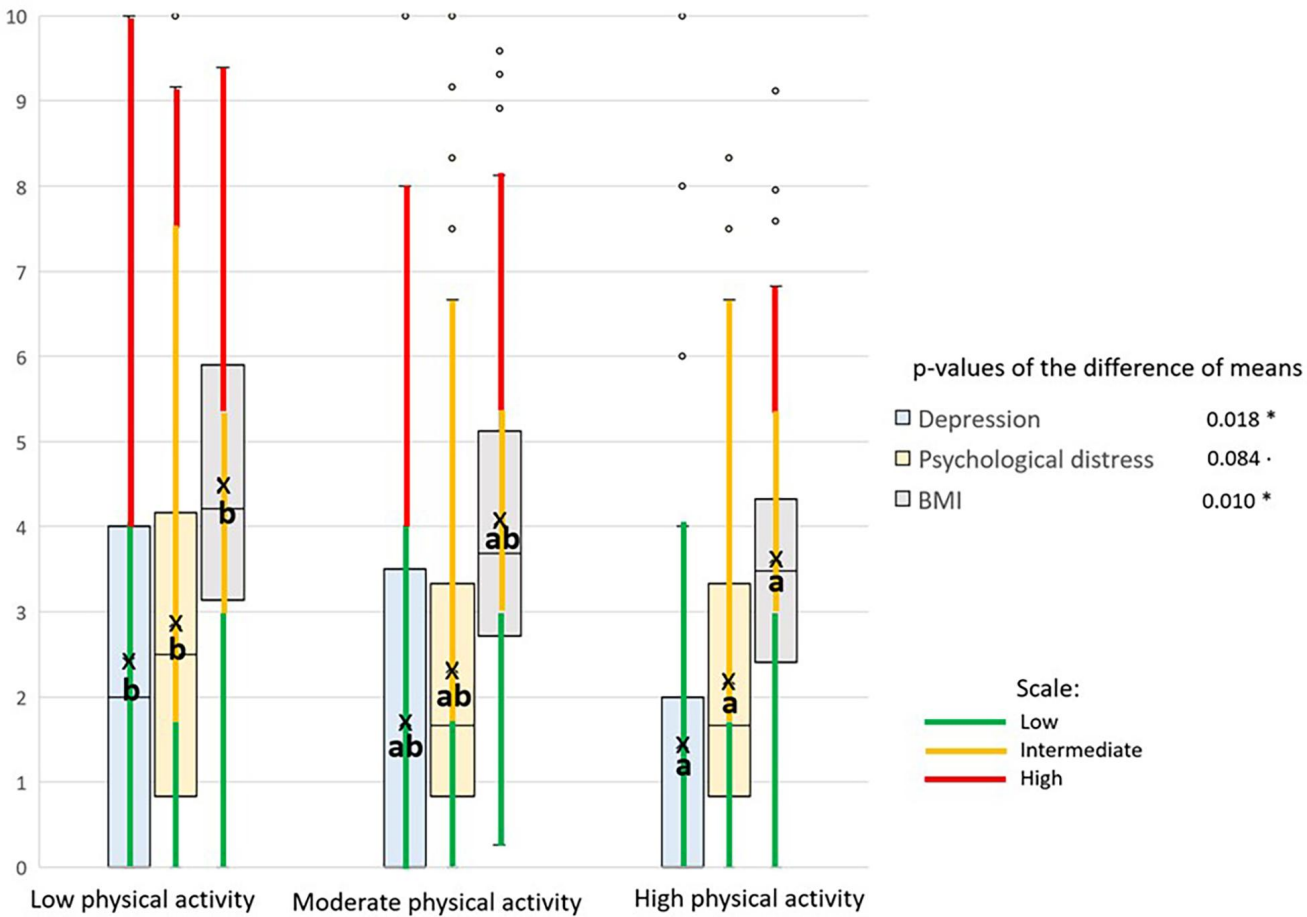
Box plots of the variables with inverse association with physical activity (low, moderate, or high), represented in a zero-to-ten scale. The categories of each scale are shown along the whisker, using the same color coding presented in Table 1. Means are represented by ‘x' inside the box (ANOVA *p*-values: · *p*-value < 0.1; *: *p*-value < 0.05). In Tukey's multiple comparisons, ‘a' is significantly less than ‘b,' and ‘ab' is not significantly different to either ‘a' or ‘b’.

Figures 2, 3 use box and whisker plots to compare means of the variables that showed significant differences between at least two categories of PA. For each variable, the mean labelled ‘a’ is statistically lower than that labelled ‘b’ depending on the level of PA. The mean labelled ‘ab’ is not significantly different from those labelled ‘a’ or ‘b.’ Moreover, the categories of each variable are shown along the whiskers using the same color coding presented in Table 1. It should be noted that purpose in life was not categorized.

Figures 2, 3 show differences in mean psychosocial scores across PA categories. Resilience, sense of coherence, and social isolation differed between low and moderate PA groups, whereas depression and psychological distress differed between low and high PA groups. Figures 2, 3 also show the boxes of variables arranged in ascending order. In Figure 2, regardless of PA, means are higher for psychosocial variables, such as purpose in life and resilience, while a low level of PA is associated with lower scores for all the variables (direct association). Similarly, in Figure 3 all the variables’ means are lower irrespective of PA while a low level of PA is significantly associated with higher scores for depression, psychological distress, and body mass index (inverse association).

## 4 Discussion

This study examines the association between PA and mental health risk in older adults, with a focus on psychosocial factors related to PA levels. These results are consistent with findings from other cross-sectional studies reporting associations between activity levels and psychosocial profiles (37, 38). Given that PA assessment relied on the IPAQ-SF—a self-report instrument vulnerable to recall and social desirability biases that may overestimate activity—results should be interpreted cautiously. The observed patterns of association —moderate PA with resilience and sense of coherence, high PA with depressive symptoms—suggest a graded pattern across activity categories; however, longitudinal evidence would be required to determine directionality or causality.

In this study, mental health risk is operationalized as a single composite ordinal variable, integrating indicators of stress and depression risk. This approach did not entail the evaluation of each condition as an independent binary outcome or the temporal sequence between them. However, some longitudinal evidence suggests that stress can occur prior to depression in certain individuals. Chronic psychological stress has also been associated with inflammatory markers in prospective studies, which have been proposed as possible links between stress and depressive symptoms (39). Cognitive models propose that stress is linked to negative thinking patterns and emotional vulnerability, which are associated with higher likelihood of depression (5). A substantial proportion of depressive episodes have been reported following major life events, with stress frequently identified as a contributing factor. In addition, a recent analysis of the pairwise comorbidity of different types of mental disorders has reported that the onset of one mental disorder tends to co-occur with the later appearance of a second condition up to 15 years later (40). Together, these findings suggest that stress is an important factor associated with the emergence of depressive disorders. According to data in the literature, biological, developmental, psychological, and sociodemographic factors are associated with variation in the relationship between stress and depression. The prevalence of depression and stress is higher among women and tends to increase with age (2), while marital status has been related to differences in the prognosis in adults previously diagnosed with depression (41). We calculated a prevalence of mental health risk of 52% in our sample and observed lower mental health risk among married participants than among those separated, widowed or single. We found no association between sex and mental health risk. Among the 16 variables statistically associated with mental health risk, most were psychosocial and lifestyle factors. When participants were profiled based on their screening test results, higher or lower mental health risk corresponded to less favorable or more favorable score patterns, respectively. However, the cross-sectional design does not allow inference about temporality, directionality, or causality between the screened variables and mental health risk.

Considerable evidence documents associations between loneliness and mental health risk (42). People who feel lonely are more likely to report chronic health problems, including higher levels of depression, anxiety, cognitive impairment, and physical health difficulties. However, other factors, such as personality and motivational constructs including sense of coherence and resilience, may also be relevant. On the one hand, a high sense of coherence has been associated with better perceived ability to manage resources and cope with stressful circumstances. It has also been linked to lower levels of cognitive dysfunction, dependence, frailty, and mortality (43). On the other hand, higher resilience is associated with more effective stress management. Resilience has been reported to increase following exposure to manageable stressors that do not exceed individual coping thresholds (44).

PA may be viewed as a modifiable behavior that can be tailored to a participant's mood, psychosocial, and lifestyle profile (45). Several studies have documented an association between PA and symptoms of depression or psychological stress in different populations (46–48). Walking, jogging, strength training, and yoga are commonly used and well-tolerated exercise modalities in programs aimed at managing depressive symptoms (49). Greater purpose in life also correlates with higher levels of PA over four years of follow-up (50), while moderate to high levels of PA are associated with lower likelihood of social isolation by up to 30% (51). Participation in sports and exercise groups is associated with higher PA and greater social support, factors that have been linked to lower reported loneliness (52). Regular PA has been associated with greater persistence and adaptability in uncomfortable situations. PA has been associated with higher psychological resilience, and tolerance of exercise has been proposed as a potential intermediary factor in this relationship (34, 53). Overall, PA appears to co-occur with lower mental health risk. We highlight resilience and a sense of coherence because in our cross-sectional data these factors showed associations with moderate PA, whereas depression showed associations with high PA. Integrating psychosocial profiling into routine healthcare may inform the development of personalized PA approaches. Our findings indicate that comprehensive assessments may be implementable in community-based settings, where pharmacists—among the most accessible healthcare professionals— could potentially contribute to psychosocial-informed PA recommendations. A recent review of PA interventions in community pharmacies (54) found that most initiatives remain nonspecific and largely target cardiovascular risk, highlighting a potential gap in structured and individualized approaches. Incorporating psychosocial assessment into pharmacy practice could contribute to moving PA beyond generic lifestyle advice toward more tailored recommendations. Under an individualized recommendation framework, PA could encompass: (i) target outcomes (e.g., depressive symptoms), (ii) suggested activity level (moderate to high), (iii) individual risk factors (age, social vulnerability), and (iv) monitoring parameters (psychosocial assessments, mental health screening). This approach may be advantageous given the minimal low adverse effects of PA and its reported association with a broad range of health benefits (55). Although convenience sampling precludes extrapolation to the general population, the findings likely reflect characteristics of this specific clinical cohort and may offer practical relevance for patients seen in this setting. This approach is not without barriers, and adherence is a significant obstacle. Factors associated with PA adherence in older adults include age and motivation,. while current clinical recommendations for PA are often broad and may not account for individual limitations (30, 56). Identifying individuals at risk for stress and depression would likely require routine screening for these conditions.

### 4.1 Strengths and limitations

This cross-sectional design prevents determination of causal relationships regarding PA and mental health outcomes, though our findings may provide a foundation for prospective longitudinal studies to examine directionality. A key strength of this study is the use of face-to-face interviews (averaging approximately 69 min) conducted by trained pharmacists, which likely improved standardized questionnaire administration, allowed clarification when needed, and may have reduced missing or misunderstood responses, an approach especially relevant in older adults.

The IPAQ-SF focuses on intensity rather than activity type, limiting information about specific exercise modalities (resistance, aerobics, balance) and their associations with outcomes. The use of this instrument may lead to misclassification and overestimation. However, the IPAQ-SF is widely validated and considered feasible for use in preventive, community-based settings such as pharmacies and primary care centers, where objective measures are not easily deployable. Its low time burden and comparability across studies support its use in research examining behavioral and preventive health factors in large populations. Future research should examine associations between specific exercise modalities and the psychosocial outcomes identified.

The study sample was recruited using a convenience sampling method. This may not be representative of older adult populations; however, recruitment through highly accessible points of care may increase the contextual relevance of the findings for real-world preventive settings. Moreover, data collection during the post-COVID-19 phase may have influenced psychosocial responses. Future studies should test these psychosocial-PA associations in diverse environments and examine whether longitudinal interventions tailored to individual psychosocial profiles are associated with changes in mental health outcomes.

## 5 Conclusion

This study characterizes associations between PA and mental health outcomes in older adults, with individual psychosocial factors varying systematically across PA levels. Our findings suggest that psychosocial profiling may be useful to consider when developing more personalized PA recommendations compared to generic approaches. The observed associations—moderate PA with higher resilience and sense of coherence, high PA with lower depressive symptoms—are consistent with those described in the literature, though prospective studies are needed to establish temporal relationships and explore potential causal mechanisms. The community-based assessment approach suggests that incorporating psychosocial profiling into routine healthcare settings, particularly through accessible facilities like community pharmacies. Such integration could inform PA recommendations that account for individual psychosocial characteristics.

Future research should consider: (i) systematic psychosocial assessment tools for individual profiling, (ii) further exploration of associations between activity levels and specific outcomes, (iii) healthcare provider training in psychosocial-informed PA counseling-approaches, and (iv) digital health technologies to support personalized recommendations and monitor adherence patterns. Understanding psychosocial factors associated with PA levels may help guide future efforts toward more personalized health recommendations for mental health promotion in aging populations. However, the temporal sequence and mechanisms underlying the associations between PA and mental health outcomes require evaluation through prospective and experimental studies. Cross sectional findings should be interpreted as descriptive associations that may inform screening and counseling practices rather than as evidence supporting PA as a therapeutic intervention for mental health conditions.

## Data Availability

The raw data supporting the conclusions of this article will be made available by the authors, without undue reservation.
