# Supplementary material for: Physical activity and psychosocial profiles associated with mental health risk in community-dwelling older adults: a cross-sectional study
Source: Front Sports Act Living. 2026 Mar 25;8:1702851. doi: 10.3389/fspor.2026.1702851 (PMC13058800; doi:10.3389/fspor.2026.1702851)
Supplement: Supplementary file 1 [file Table1.docx]

Supplementary Material

# Supplementary Table 1

Table S1. Summary of assessment tools: no. of items, clinical cut-offs, and key validation references.

| **Data Collection Structure** | | | | | **Results** |
| --- | --- | --- | --- | --- | --- |
| **Variable** | **Validated test (Reference) and/or categories** | **Definition** | **Type of answer** | **No. of items** | **Mean ± SD** |
| **a. Sociodemographic Factors** | | | | | |
| Age | Total | “Older adults" refers to individuals over 65, but also apply to those over 50 in contexts like healthy ageing and chronic disease. | Grouped quantitative | 1 | 73.3 ± 10.5 |
|  | [50, 59] |  |  |  | 55.6 ± 2.6 |
|  | [60, 69] |  |  |  | 64.6 ± 3.0 |
|  | [70, 79] |  |  |  | 75.2 ± 2.8 |
|  | [80, 89] |  |  |  | 85.2 ± 3.8 |
| Sex | Women |  | Dichotomic | 1 |  |
|  | Men |  |  |  |  |
| Marital status | Married |  | Multiple choice single answer | 1 |  |
|  | Widow |  |  |  |  |
|  | Separated/divorced |  |  |  |  |
|  | Single |  |  |  |  |
| **b. Cognitive Impairment (CI)** | | | | | |
| CI risk  (At least one positive test) | Memory Impairment Screen [0, 8]  (1) | Episodic memory (four words), a distractor task, and a recall test with or without cues. | Free and cued recall | 8 | 6.9 ± 1.8 |
|  | CI risk [0, 4] |  |  |  | 2.4 ± 1.4 |
|  | No CI risk (4, 8] |  |  |  | 7.4 ± 0.9 |
|  | Short Portable Mental State Questionnaire [0–10] (2) | Questions covering orientation, calculation, memory, and general knowledge. | Brief responses to factual questions | 10 | 1.0 ± 1.0 |
|  | CI risk [3–4, 10] |  |  |  | 3.9 ± 1.0 |
|  | No CI risk [0, 3–4) |  |  |  | 0.7 ± 0.8 |
|  | Semantic Verbal Fluency [0, +∞) (3) | Name as many animals as possible in 60 seconds. | Free verbal production | 1 | 17.8 ± 6.8 |
|  | CI risk [0, 10] |  |  |  | 7.5 ± 1.6 |
|  | No CI risk [10, +∞) |  |  |  | 18.5 ± 6.5 |
| **c. Meaning in Life** | | | | | |
| Sense of coherence | Orientation to Life Questionnaire (OLQ-13)  [13, 91] (4) | Assessment of ability to see life as comprehensible, manageable, and meaningful | 7-point Likert scale | 13 | 68.5 ± 11.8 |
|  | Low [13, 64[ |  |  |  | 54.0 ± 7.7 |
|  | Intermediate [64, 80[ |  |  |  | 72.0 ± 4.4 |
|  | High [80, 91] |  |  |  | 83.2 ± 3.0 |
| Purpose in life (PiL) | PiL subscale of Ryff’s Well-Being Scale [6, 36] (5) | Assessment of sense of meaning, direction, and purpose in life. | 6-point Likert scale | 6 | 29.9 ± 5.2 |
| **d. Psychosocial Factors** | | | | | |
| Resilience | Brief Resilient Coping Scale (BRCS) [4, 20] (6) | Assessment of adaptive and resilient coping with stress. | 5-point Likert scale | 4 | 15.8 ± 4.2 |
|  | Low [4, 13) |  |  |  | 10.0 ± 2.1 |
|  | Intermediate [13, 17] |  |  |  | 15.3 ± 1.3 |
|  | High (17, 20] |  |  |  | 19.4 ± 5.0 |
| Psychological distress | Patient Health Questionnaire-4 (PHQ-4) [0–12] (7) | Screening depression and anxiety (PHQ-2, GAD-2). | 4-point Likert scale | 4 | 2.9 ± 2.7 |
|  | None [0, 2] |  |  |  | 1.0 ± 0.9 |
|  | Mild [3, 5] |  |  |  | 3.8 ± 0.7 |
|  | Moderate [6, 8] |  |  |  | 6.7 ± 0.9 |
|  | Severe [9, 12] |  |  |  | 10.5 ± 1.1 |
| Loneliness | UCLA-3 [3, 9] (8) | Assessment of perceived loneliness through feelings of isolation, lack of companionship, and disconnection. | 3-point Likert scale | 3 | 3.9 ± 1.4 |
|  | Loneliness [6, 9] |  |  |  | 7.0 ± 1.1 |
|  | No loneliness [3, 6) |  |  |  | 3.5 ± 0.8 |
| Social isolation | Lubben Social Network Scale (LSNS-6) [0, 30] (9) | Assessment of frequency and closeness of interactions with family and friends. | 5-point Likert scale | 6 | 19.1 ± 5.2 |
|  | Social isolation [0, 12] |  |  |  | 9.7 ± 2.2 |
|  | No social isolation (12, 30] |  |  |  | 20.2 ± 4.2 |
| **e. Health problems** | | | | | |
| Hypertension | Yes [systolic BP > 140 mmHg–diastolic BP > 90 mmHg] (10) | | All measurements were obtained from analytical results or by using the WHO STEPwise approach. | 3 |  |
|  | No [systolic BP 140 mmHg–diastolic BP 90 mmHg] | |  |  |  |
| Hypercholesterolemia | Yes [CT > 200 mg/dL–CLDL > 100 mg/dL–CHDL 35–40 mg/dL] (11) | |  |  |  |
|  | No [CT 200 mg/dL–CLDL 100 mg/dL–CHDL > 35–40 mg/dL] | |  |  |  |
| Diabetes | Yes [blood glucose > 126 mg/dL–HbA1c > 6.5%] (12) | |  |  |  |
|  | No [blood glucose 126 mg/dL–HbA1c < 6.5%] | |  |  |  |
| Smoking habit | Non-smoker | | Self-reported | 1 |  |
|  | Former smoker | |  |  |  |
|  | Smoker | |  |  |  |
|  | Passive smoker | |  |  |  |
| Risk of cardiovascular disease | ERICE scale [1, 84] (13) | 10-year cardiovascular risk using age, sex, blood pressure, smoking, diabetes, cholesterol, and antihypertensive treatment. | Clinical objective variables | 7 | 27.9 ± 16.3 |
|  | Low [1, 5) |  |  |  | 2 ± 1.7 |
|  | Mild [5, 9] |  |  |  | 7.1 ± 1.7 |
|  | Moderate [10, 14] |  |  |  | 12.2 ± 1.3 |
|  | Moderate–high [15, 19] |  |  |  | 17.8 ± 1.1 |
|  | High [20, 29] |  |  |  | 23.3 ± 2.0 |
|  | Very high [30, 84] |  |  |  | 43.6 ± 10.6 |
| Dependency | Independent P-ADL and I-ADL | P-ADL - physical autonomy; I-ADL - social and cognitive autonomy. | Dichotomous | 11 |  |
|  | Dependent |  |  |  |  |
| Chronic pain | VAS scale [0, 10] | Subjective pain intensity. | 10-point Likert scale | 1 | 3.7 ± 2.8 |
|  | No pain [0] |  |  |  | 0 |
|  | Mild [1, 3] |  |  |  | 2.2 ± 0.7 |
|  | Moderate [4, 6] |  |  |  | 5.1 ± 0.8 |
|  | Severe [7, 8] |  |  |  | 7.4 ± 0.5 |
|  | Excruciating [9, 10] |  |  |  | 9.4 ± 0.5 |
| **f. Lifestyle** | | | | | |
| Anthropometry | Body Mass Index (kg/m2) (14) | Weight and height (kg/m²). | WHO STEPwise approach | 2 | 27.2 ± 4.1 |
|  | Normal weight [18.5, 25) |  |  |  | 22.8 ± 1.6 |
|  | Overweight [25, 30) |  |  |  | 27.1 ± 1.4 |
|  | Obese [30, +∞) |  |  |  | 33.0 ± 2.3 |
| Cognitive reserve | Cognitive Reserve Questionnaire (CRQ) [0, 25] (15) | Assessment of education, occupation, language learning, and reading habits. | Multiple choice single answer | 8 | 11.3 ± 5.2 |
|  | Low [0, 6] |  |  |  | 4.2 ± 1.7 |
|  | Intermediate–low [7, 9] |  |  |  | 8.0 ± 0.8 |
|  | Intermediate–high [10, 14] |  |  |  | 11.8 ± 1.4 |
|  | High [15, 25] |  |  |  | 17.6 ± 1.9 |
| Nutrition | Mediterranean Diet Adherence Score (MeDAS) [0, 14] (16) | Assessment of adherence to the Mediterranean diet through 12 questions on food frequency and 2 on dietary habits. | Dichotomous | 14 | 9.1 ± 2.2 |
|  | Low [0, 6] |  |  |  | 5.5 ± 0.7 |
|  | Intermediate [7, 9] |  |  |  | 8.1 ± 0.8 |
|  | High [10, 14] |  |  |  | 11.1 ± 1.1 |
|  | Mini Nutritional Assessment (MNA) [0–14] (17) | Assessment of nutritional status, especially in older adults. | Multiple choice single answer | 6 | 12.1 ± 1.8 |
|  | Risk of malnutrition [0, 12) |  |  |  | 9.9 ± 1.3 |
|  | Normal nutrition [12, 14] |  |  |  | 13.2 ± 0.8 |
| Physical activity | Short version of the International Physical Activity Questionnaire (IPAQ-SF) (MET-minutes per day) (18) | Assessment of physical activity levels and sitting time over the past seven days. | Self-reported | 7 | 2571.6 ± 2807.9 |
|  | Low |  |  |  | 379.3 ± 433.4 |
|  | Moderate |  |  |  | 1633.6 ± 690.1 |
|  | High |  |  |  | 5679.6 ± 3105.0 |
| Sleep | Jenkins Sleep Scale (JSS) [0, 20]  (19) | Assessment of sleep quality and disturbances. | 6-point Likert scale | 4 | 7.3 ± 5.2 |
|  | Sleep disorder [12, 20] |  |  |  | 15.1 ± 2.8 |
|  | No sleep disorder [0, 12) |  |  |  | 5.2 ± 3.3 |
|  | STOP-Bang Questionnaire [0, 8]  (20) | Assessment of obstructive sleep apnea risk. | Dichotomous | 8 | 3.0 ± 1.5 |
|  | Low [0, 2] |  |  |  | 1.6 ± 0.5 |
|  | Intermediate [3–4] |  |  |  | 3.4 ± 0.5 |
|  | High [5, 8] |  |  |  | 5.4 0.7 |

Numerical results are described as means and standard deviations (mean ± SD). Abbreviations: BP: blood pressure; CT: total cholesterol; CLDL: low-density lipoprotein cholesterol; CHDL: high-density lipoprotein cholesterol; HbA1c: hemoglobin A1c; P-ADL: personal activities of daily living; I-ADL: instrumental activities of daily living and VAS: visual analogic scale.

**References**

1. Buschke H, Kuslansky G, Katz M, Stewart WF, Sliwinski MJ, Eckholdt HM, Lipton RB. Screening for dementia with the memory impairment screen. *Neurology* (1999) 52:231–238. doi: 10.1212/wnl.52.2.231

2. Pfeiffer E. A short portable mental status questionnaire for the assessment of organic brain deficit in elderly patients. *J Am Geriatr Soc* (1975) 23:433–441. doi: 10.1111/j.1532-5415.1975.tb00927.x

3. Carnero Pardo C, Lendínez González A. Utilidad del test de fluencia verbal semántica en el diagnóstico de demencia. *Rev Neurol* (1999) 29:709. doi: 10.33588/rn.2908.99233

4. Antonovsky A. The structure and properties of the sense of coherence scale. *Soc Sci Med* (1993) 36:725–733. doi: 10.1016/0277-9536(93)90033-Z

5. Ryff CD, Keyes CLM. The structure of psychological well-being revisited. *J Pers Soc Psychol* (1995) 69:719. doi: 10.1037/0022-3514.69.4.719

6. Sinclair VG, Wallston KA. The development and psychometric evaluation of the Brief Resilient Coping Scale. *Assessment* (2004) 11:94–101. doi: 10.1177/1073191103258144

7. Kroenke K, Spitzer RL, Williams JBW, Löwe B. An ultra-brief screening scale for anxiety and depression: the PHQ–4. *Psychosomatics* (2009) 50:613–621. doi: 10.1016/s0033-3182(09)70864-3

8. Hughes ME, Waite LJ, Hawkley LC, Cacioppo JT. A short scale for measuring loneliness in large surveys: Results from two population-based studies. *Res Aging* (2004) 26:655–672. doi: 10.1177/0164027504268574

9. Lubben J, Blozik E, Gillmann G, Iliffe S, Von Kruse WR, Beck JC, Stuck AE. Performance of an abbreviated version of the lubben social network scale among three European community-dwelling older adult populations. *Gerontologist* (2006) 46:503–513. doi: 10.1093/geront/46.4.503

10. Kreutz R, Brunström M, Burnier M, Grassi G, Januszewicz A, Muiesan ML, Tsioufis K, de Pinho RM, Albini FL, Boivin J-M, et al. 2024 European Society of Hypertension clinical practice guidelines for the management of arterial hypertension. *Eur J Intern Med* (2024) 126:1–15. doi: 10.1016/j.ejim.2024.05.033

11. Mach F, Baigent C, Catapano AL, Koskinas KC, Casula M, Badimon L, Chapman MJ, De Backer GG, Delgado V, Ference BA, et al. 2019 ESC/EAS Guidelines for the management of dyslipidaemias: Lipid modification to reduce cardiovascular risk. *Eur Heart J* (2020) 41:111–188. doi: 10.1093/eurheartj/ehz455

12. Cosentino F, Grant PJ, Aboyans V, Bailey CJ, Ceriello A, Delgado V, Federici M, Filippatos G, Grobbee DE, Hansen TB, et al. 2019 ESC Guidelines on diabetes, pre-diabetes, and cardiovascular diseases developed in collaboration with the EASD. *Eur Heart J* (2020) 41:255–323. doi: 10.1093/eurheartj/ehz486

13. Gabriel R, Brotons C, Tormo MJ, Segura A, Rigo F, Elosua R, Carbayo JA, Gavrila D, Moral I, Tuomilehto J, et al. The ERICE-score: the new native cardiovascular score for the low-risk and aged mediterranean population of Spain. *Rev Española Cardiol (English Ed* (2015) 68:205–215. doi: 10.1016/j.rec.2014.03.019

14. World Health Organization. WHO STEPS surveillance manual: the WHO STEPwise approach to chronic disease risk factor surveillance / Noncommunicable Diseases and Mental Health. (2005) https://iris.who.int/handle/10665/43376

15. Rami González L, Valls Pedret C, Bartrés Faz D, Caprile Elola-Olaso C, Solé Padullés C, Castellví Sampol M, Olives Cladera J, Bosch Capdevila B, Molinuevo Guix JL. Cuestionario de reserva cognitiva. Valores obtenidos en población anciana sana y con enfermedad de Alzheimer. *Rev Neurol* (2011) 52:195. doi: 10.33588/rn.5204.2010478

16. Ferreira-Pêgo C, Nissensohn M, Kavouras SA, Babio N, Serra-Majem L, Águila AM, Mauromoustakos A, Pérez JÁ, Salas-Salvadó J. Beverage intake assessment questionnaire: relative validity and repeatability in a Spanish population with metabolic syndrome from the PREDIMED-PLUS study. *Nutrients* (2016) 8:475. doi: 10.3390/nu8080475

17. Vellas B, Villars H, Abellan G, Soto ME, Rolland Y, Guigoz Y, Morley JE, Chumlea W, Salva A, Rubenstein LZ, et al. Overview of the MNA® - Its history and challenges. *J Nutr Heal Aging* (2006) 10:456–465.

18. Roman-Viñas B, Serra-Majem L, Hagströmer M, Ribas-Barba L, Sjöström M, Segura-Cardona R. International physical activity questionnaire: reliability and validity in a Spanish population. *Eur J Sport Sci* (2010) 10:297–304. doi: 10.1080/17461390903426667

19. Jenkins CD, Stanton BA, Niemcryk SJ, Rose RM. A scale for the estimation of sleep problems in clinical research. *J Clin Epidemiol* (1988) 41:313–321. doi: 10.1016/0895-4356(88)90138-2

20. Chung F, Abdullah HR, Liao P. STOP-bang questionnaire a practical approach to screen for obstructive sleep apnea. *Chest* (2016) 149:631–638. doi: 10.1378/chest.15-0903
